# Three-Dimensional Electrosorption for Pharmaceutical Wastewater Management and Sustainable Biochar Regeneration

**DOI:** 10.3390/molecules30071435

**Published:** 2025-03-24

**Authors:** Nuria Bernárdez-Rodas, Emilio Rosales, Marta Pazos, Óscar González-Prieto, Luis Ortiz Torres, M. Ángeles Sanromán

**Affiliations:** 1 CINTECX, Bioengineering and Sustainable Processes Group, Chemical Engineering Department, Universidade de Vigo, Campus Lagoas-Marcosende, 36310 Vigo, Spain; nuria.bernardez@uvigo.gal (N.B.-R.); emiliorv@uvigo.gal (E.R.); mcurras@uvigo.gal (M.P.); 2 Hydro-Forestry Geomodeling Research Group, School of Forestry Engineering, University of Vigo, 36005 Pontevedra, Spain; osgonzalez@uvigo.gal (Ó.G.-P.); lortiz@uvigo.gal (L.O.T.)

**Keywords:** biochar, three-dimensional electrosorption, sulfamethizole, fluoxetine, mechanisms, regeneration

## Abstract

The adsorption capacity of a biochar (BC) obtained from pine wood residues was evaluated for its ability to remove two pharmaceuticals: fluoxetine (FLX) and sulfamethizole (SMZ). The material showed promising results in FLX removal, but a limited capacity in the case of SMZ. In order to improve these results, BC surface modifications were made by doping with nitrogen, as well as using acid, basic and electrochemical treatments. A three-dimensional electrosorption treatment proved to be the most effective, increasing the adsorption rate from 0.45 to 13.46 mg/g after evaluating different operating conditions, such as the electrodes used or the BC dosage. Consecutive cycles of BC use were performed through desorption and electro-regeneration techniques to test its capacity for reuse, and it was observed that application in the 25 mA electric field increased the useful life of the material. Finally, the effect of ionic strength was studied, highlighting that the presence of ions did not significantly affect the efficiency of SMZ removal, although a slight increase was observed at a high ion concentration, probably due to a salinization effect.

## 1. Introduction

The pollution of water bodies by the presence of emerging pollutants poses an increasingly serious threat to human health and the environment [1]. These pollutants include pharmaceutical compounds such as antibiotics (sulfamethizole) and antidepressants (fluoxetine) which cannot be fully metabolized by the human body [2]. Consequently, these residues are accumulated in wastewater treatment plants, where they are not efficiently removed and are eventually discharged into aquatic environments [3,4,5]. These compounds, which are being used more frequently, are characterized by their high persistence in the environment, and their accumulation can lead to genotoxic, mutagenic and ecotoxicological effects [6,7].

Fluoxetine (FLX) is an antidepressant that is extensively prescribed and taken to treat depression and anxiety disorders, becoming one of the most present in aquatic environments [8,9,10]. Numerous studies have confirmed the high persistence of this pollutant by measuring its concentration in wastewater treatment plant effluents: in Costa Rica, concentrations of up to 100 ng/L were detected, while in Canada, levels between 7.6 and 20 ng/L were found, according to the treatment system and population capacity [11]. Traces of this pollutant were also identified in Europe, reaching 29 ng/L in the Leça River or 2 ng/L in the Douro River in Portugal [12], and reaching 0.27 ng/L in the United Kingdom [13]. These data, together with the high-risk quotient associated with FLX [14], highlight the urgency of reducing and eliminating this compound in water bodies.

A similar situation occurs in the case of antibiotics, where widespread use has significantly increased their presence in aquatic environments [15,16,17]. Within this group, sulfonamides are widely used because of their low cost and high efficacy against Gram-positive and Gram-negative bacteria [18]. However, their chemical stability makes them highly persistent, and their presence has been reported in Asian, European and African countries [19,20,21,22,23]. Sulfamethizole (SMZ) is a representative example of the sulfonamide group, which can be excreted by the body at up to 80% of the administered dose. As a result, its presence in groundwater has reached levels exceeding 300 µg/L [24], making it necessary to implement proper treatments for its removal.

The removal of pharmaceutical pollutants through economic and sustainable methods has been the aim of numerous studies, where multiple techniques for wastewater remediation have been explored, such as ozonation [25], photocatalysis [26], electrochemical oxidation [27], electro-Fenton processes and derivatives [28] or adsorption processes [29], among others.

One of the most common processes consists of the adsorption of pollutants using activated carbon (AC). However, in recent decades, this field has evolved towards the use of biosorbents, giving rise to the formation of hydrochars and BC. BC is a carbonaceous material obtained from organic biomass by slow pyrolysis, a controlled thermal decomposition in the absence of oxygen [30,31].

Beyond its use in wastewater treatment, BC has proven to be a highly effective tool in soil remediation and carbon sequestration, addressing climate change issues and contributing to achieving net-zero carbon emissions [32]. This objective implies the need to counteract the CO_2_ emitted into the atmosphere through processes that mitigate and/or eliminate the presence of this gas [33]. Furthermore, this material is also frequently employed as an energy source, reducing the emissions generated compared to the combustion of fossil waste [34].

All this, together with BC production as an effective solution for the management and valorization of organic waste such as biomass, makes it possible to align the production and use of this material with the principles of the circular economy [35,36]. In the literature, feedstocks of a diverse origin are reported for obtaining BC, including agricultural residues [37,38], sludge [39,40] or industrial waste [41]. Particularly, BCs derived from biomass waste have been very popular in pharmaceutical adsorption processes. Some examples include Guo et al. [42], with a BC derived from corn straw for tetracycline removal; Naghipour et al. [43], with a BC derived from pine cones for cefixime adsorption; or Alawa et al. [44], with the development of a BC from wheat straw residues for ciprofloxacin elimination.

In addition to being a more sustainable and eco-friendly option, BC offers significant cost benefits, as its acquisition price can vary between $350 and $1200 per ton, while AC can reach up to $1700 [45].

The high effectiveness of this material is directly related to parameters such as its porosity, surface area and functional groups, so many studies have tried to enhance these properties though a variety of modification strategies [46]. The modification of BC surface properties by nitrogen doping or acid and alkaline treatments has been a recurring approach in the literature [47,48,49]. Additionally, electrochemical techniques have been used to achieve greater efficiency in adsorption processes, specifically by electro-assisted adsorption or electrosorption [50].

Electrosorption is based on the use of a low electric potential field applied to enhance the adsorption of ions or charged molecules onto the adsorbent surface by polarization forming double-layer electrical capacitors, which makes it a useful process for improving the efficiency and selectivity of the process [51]. Although electrosorption processes were originally focused on water desalination [52,53], several studies in recent years have explored their application for heavy metal removal from aqueous solutions, as well as for removing certain organic pollutants [54,55]. However, the application of electrosorption assays in pharmaceutical removal, particularly in three-dimensional (3D) polarization systems, is still limited. Compared to a conventional two-dimensional (2D) system, 3D electrosorption allows a larger active surface area to be achieved, providing the interaction between the adsorbent and adsorbate and optimizing the availability of the active sites to the pollutants in the adsorbent [56]. For example, Alighardashi et al. [57] developed a 3D electrosorption system that successfully increased the removal rate of carbamazepine to 89.8% compared to the 29.7% that was achieved in a 2D system. Wu et al. [58] showed similar behavior, using dye instead of a pharmaceutical as model pollutant. In this case, the removal rate of Reactive brilliant red X-3B increased from 20% to 95% with the application of a 3D electrode system. These approaches demonstrate the effectiveness of 3D systems in pollutant removal, which may overcome some of the limitations of 2D systems. However, studies on this technique are still incipient, and further research is needed to better understand its feasibility and optimization, as well as to evaluate its applicability in wastewater treatment on a larger scale.

This research assessed the adsorption capacity of fluoxetine (FLX) and sulfamethizole (SMZ) using BC produced from pine wood. The adsorbent material selection was based on the use of waste biomass from a failed local plantation, with failure caused by inadequate silvicultural conditions [59]. Likewise, different modification methods to enhance the process were investigated. Hydrothermal, acid and basic treatments, as well as a 3D electrosorption approach where an electric field was applied on the BC dispersed in an electrolytic medium, were accomplished to improve the BC adsorption capacity. Other aspects of the viability of BC were also evaluated, such as its capacity for reuse, subjecting it to different regeneration techniques by desorption and electro-regeneration. Finally, an approximation to more realistic wastewater environments was conducted by evaluating the impact of ionic strength. Thus, this work aimed to contribute to the development of more efficient and sustainable solutions for treating pharmaceuticals in wastewater.

## 2. Results and Discussion

### 2.1. BC Characterization

**Figure 1:**
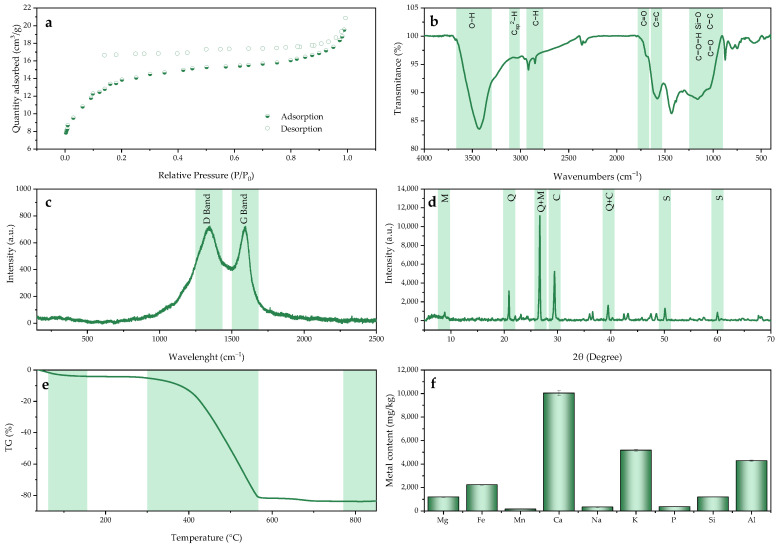
Physicochemical characterization of BC through (**a**) BET; (**b**) FTIR; (**c**) XRD; (**d**) Raman; (**e**) TGA and (**f**) ICP-OES.

The determination of surface properties and porosity is highly useful when evaluating the viability of an adsorbent for an adsorption process. Previous studies revealed a positive correlation between the material structure and its adsorption performance [60]. Thus, it was suggested that the adsorption rate was favored by the external surface area and mesoporous pore volume, while the adsorption capacity was intrinsically associated with the microporous surface area and pore volume. The Brunauer–Emmet–Teller analysis (BET), based on the physical adsorption of N_2_ onto the material at a low temperature, provided detailed information on the material’s surface area and pore volume. In Figure 1a, the adsorption–desorption isotherms for the BC are shown, which correspond to a combination of Type II and IV isotherms based on the classification established by the IUPAC [61], indicating the co-existence of micropores and mesopores on the material’s surface. A Type H4 hysteresis was also observed, generally associated with capillary condensation in mesoporous structures. This lack of closure in the hysteresis loop may have been attributed to the swelling of the material during adsorption or the retention of nitrogen molecules [62,63]. The remaining parameters related to BC surface morphology are listed in Table 1. These results are consistent with the surface area obtained by Yargicoglu et al. [64] in a BC production process through the slow pyrolysis from pine wood.

The presence of the functional groups on the BC surface was identified based on the Fourier transform infrared spectroscopy spectrum (FTIR) shown in Figure 1b, which provides information about the structure and chemical properties of the material. A broad band at 3430 cm^−1^ was observed, associated with the stretching of OH bonds [65,66,67], while a small peak at 3676 cm^−1^ could be attributed to the stretching of a hydroxyl group (OH^−^) [68]. The literature suggests that the presence of these groups is associated with the formation of hydrogen bonds in the adsorption of pollutants [69,70]. The presence of aromatic rings was confirmed by the band at 3036 cm^−1^, associated with the Csp^2^-H bond stretching typical of these groups [67,71], and which enhance the BC’s properties for adsorption processes through π-π interactions. Small bands were also observed between 2849 and 2955 cm^−1^, which are often associated in the literature with C-H bond stretching, specifically with aliphatic Csp^3^-H bonds [65,66,71,72] and with the bending modes of CH_2_ and CH_3_ hydrocarbons in the range of 1385 to 1432 cm^−1^ [72]. Possible C=O bond stretches from different chemical environments were located in the 1700–1740 cm^−1^ range [66,73,74], while the C=C bond stretching between 1618 and 1480 cm^−1^ also indicated the presence of aromatic rings [66,71,73]. Bands between 900 and 1250 cm^−1^ could have been due to Si-O, C-C, C-O and C-OH bond stretches [73], which were also involved in adsorption process efficiency [75,76]. Bands at 470, 570, 748, 800 and 1036 cm^−1^ could have been attributed to Si-O bond vibrations in silicate minerals [65]. Bands characteristic of the carbonate group (CO_3_^2^^−^), and more specifically, calcium carbonate, were detected at wavelengths of 1794, 1432, 874 and 714 cm^−1^. Moreover, the band at 874 cm^−1^ could be attributed to out-of-plane CH bending in aromatic rings [65].

The X-ray Diffraction (XRD) analysis shown in Figure 1c allows for the crystallinity study of the compounds present in BC, providing information on the degree of carbonization, the presence of minerals and the structure of the inorganic phases. If the material was crystalline in nature, the detected peaks were well defined, while broad peaks indicated the presence of an amorphous structure [77]. Adsorption processes can lead to changes in the molecular and crystalline structure of BC, so understanding their nature can be useful for optimizing adsorption performance [78]. The identification of distinct peaks suggested the presence of coexisting inorganic components, primarily quartz, located at 20.89°, 26.67°, 39.48°, 50.14° and 59.98°. Muscovite was located at 8.80° and combined with quartz at 26.67°. Calcite was also detected independently at 29.42°, as well as detected combined with quartz at 39.48°. The characteristic peaks of quartz and calcite, located at 26.6° and 29.4°, respectively, have been previously reported in other BC production studies [79,80]. In BC derived from bamboo, Yadav et al. [81] also detected a diffraction peak characteristic of the presence of muscovite minerals at 8.9°. The evaluation of the crystallinity was completed by determining the degree of graphitization, obtained through Raman spectroscopy and presented in Figure 1d. This technique is based on the emission of monochromatic light from a laser, which interacts with the sample. Thus, the inelastic scattering of this light (Raman scattering) provides information on the vibrational modes of the molecules, generating a spectrum associated with the specific bonds of the analyzed material [77].

Two clearly differentiated bands were observed in the ranges of 1350–1370 cm^−1^ and 1580–1600 cm^−1^. Specifically, the band at 1350 cm^−1^, known as the “D” band, corresponded to the vibrations of sp^2^ carbon structures linked with structural defects, while the band detected at 1595 cm^−1^ has been associated with the “G” band, characteristic of in-plane vibrations in graphite carbon structures [82]. The ratio between the I_D_/I_G_ indices has been directly associated with the degree of graphitization or crystallinity [83]. Therefore, after performing the fitting using Lorentzian functions, this index was calculated, resulting in a value of 1.29, which indicated a predominantly amorphous carbon structure, with the results being in line with those reported by Nan et al. [84]. A high index indicated a high degree of disorder in the BC and/or an increase in sp^3^ defects, which could provide a larger surface area with more available functional groups and, consequently, enhance adsorption [77].

Understanding the material’s stability and response to thermal changes provides important information about its decomposition and modification of its components. Therefore, it is necessary to analyze these characteristics when synthesizing, activating and functionalizing adsorbents by thermal processes, as well as to define the operating conditions in an adsorption process [77]. The material thermal stability was analyzed using thermogravimetric analysis (TGA), with the obtained profile shown in Figure 1e, displaying two distinct stages. The first stage of mass loss occurred between 50 and 150 °C, due to the evaporation of moisture present in the sample from atmospheric humidity, where the mass was reduced by approximately 5.26%. This was followed by a stable region up to 300 °C, as the evaporation of volatile compounds like hemicellulose and cellulose during pyrolysis resulted in a thermally stable BC in this temperature range. The most significant mass reduction was observed between 300 and 550 °C, with an additional 77.63% mass loss, as the lignin began to gradually combust in this range [85]. A higher combustion rate started at 400 °C due to the oxidation of amorphous carbon [86]. In the final stage of the process, when a temperature of 800 °C was reached, the biomass loss was approximately 2%, indicating the completion of the combustion process. This profile aligns with the findings of Piersa et al. [87] in terms of the evaluation of the thermal stability of a BC obtained from pine wood.

Regarding the chemical composition of the material, the inductively coupled plasma optical emission spectrometry (ICP-OES) technique allowed for the quantification of metal content such as magnesium, iron, manganese, calcium, sodium, potassium, phosphorous, silicon and aluminum which were present in the material, with their concentrations shown in Figure 1f. Additionally, BC elemental composition was determined and presented in Table 1, showing a high degree of carbonization, with a carbon content exceeding 70%.

The oxygen content was determined according to Cárdenas-Aguiar et al. [88], based on the data obtained in the elemental analysis and the ash percentage present in the material reported by González-Prieto et al. [59].

**Figure 2:**
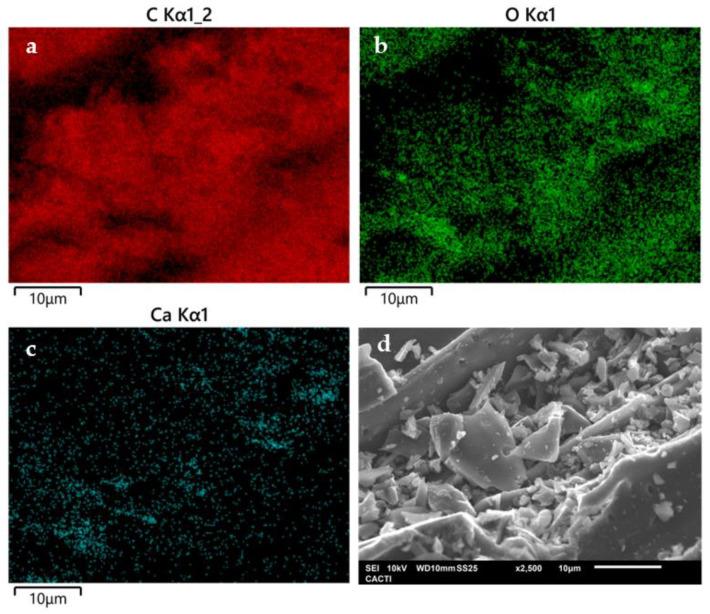
EDS mapping of (**a**) carbon, (**b**) oxygen and (**c**) Calcium present in BC. (**d**) Micrography from BC at x2500 resolution.

These results were consistent with those obtained in the SEM/EDS analysis. A predominant presence of C was found (Figure 2a), followed by O (Figure 2b) and Ca (Figure 2c), along with other less prominent compounds such as K, Mg, Si and Al, all of which are commonly found in BC derived from wood [64,89]. The micrograph in Figure 2d shows the surface morphology of the material, revealing a hierarchical structure of granular and irregular pores in shape and size on BC surface.

In addition, the pH_zpc_ of the material was determined to study the charge distribution on its surface, which is included in Table 1 with a value of 7.67. This implied that if the working solution had a more acidic pH, the surface charge of the material would be predominantly positive, while at higher pH values, the BC surface would be predominantly negative [90].

### 2.2. Cationic and Anionic Adsorption

The pH is a determining factor when evaluating the adsorption efficiency of ionizable pollutants, since the microspecies distribution is dependent on it. Thus, the behavior of BC was evaluated with contaminants that showed ionized forms with charges opposite to their respective working pH values, FLX (7.15) and SMZ (5.57). Table 2 presents the dissociation constants (pK_a_) for each compound, as well as the octanol–water partition coefficients (K_ow_), which are indicative of the hydrophobicity of the compound.

Since FLX contains an amine group in its structure, the behavior of this molecule is considered to be basic in aqueous solutions [93]. Therefore, for pH values < pK_a_ (basic), as in this case, the molecule was predominantly in a state with a net positive charge [94]. On the other hand, SMZ is considered an amphoteric molecule (type N) as it belongs to the sulfonamide group. Since these molecules contain both an acidic and a basic group, they are ionized in their cationic form when pH < pK_a_ (acidic), in their neutral form when pK_a_ (acidic) < pH < pK_a_ (basic) and in their anionic form when pK_a_ (basic) < pH [93]. Thus, it was concluded that SMZ was negatively ionized during the test. Furthermore, since in both cases, the pH was lower than pH_zpc_, the surface of the BC remained positively charged in both tests.

**Figure 3:**
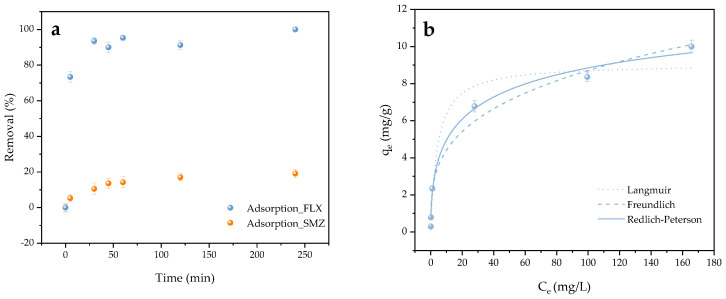
(**a**) FLX (blue) and SMZ (orange) removal achieved by BC in 4 h of contact with dose of 10 g/L and pollutant concentration of 10 mg/L; and (**b**) FLX isotherm fittings to Langmuir, Freundlich and Redlich–Peterson models.

Contact time is another important parameter in the removal efficiency of pollutants. Figure 3a shows the removal percentages achieved for each contaminant over time, calculated according to Equation (S1) (Table S1). Despite expectations, satisfactory results were obtained for FLX, reaching a removal rate of over 90% in just 30 min, which remained stable until the test was completed at 120 min. In contrast, the removal efficiency was very limited for SMZ, which reached 10% in 30 min and continued to increase until it stabilized at 17% at the end of the treatment. This behavior discarded electrostatic interactions as being the main adsorption mechanism in the process.

Other authors have reported good FLX adsorption in biochar despite the existence of electrostatic repulsion between the positive charges on the material’s surface and the protonated form of the contaminant [95]. Thus, other mechanisms such as hydrogen bonding, hydrophobic interactions or π-π electron donor–acceptor interactions could have been responsible for the good adsorption capacity of FLX by the BC. Hydrogen bond formation occurred because of the interaction between the highly electronegative fluorine atoms of FLX and the -OH groups present on the BC surface, as well as being a result of the interaction between the NH_2_ group of FLX and the oxygenated groups on the BC (-C-O and C=O). The involvement of π-π dispersive coupling occurred in response to interactions between the aromatic rings of FLX and the aromatic and π donor groups present in the BC, such as -OH groups or benzene rings. Moreover, the moderate hydrophobic nature of FLX may have also contributed to the adsorption mechanism, favoring the interaction between the BC surface and the contaminant molecules in an aqueous environment [96,97,98].

Micropore filling was proposed in previous studies as a mechanism for the adsorption of sulfonamides due to their small molecular size [99]; this is what could lead to SMZ removal. However, as previously mentioned, the available surface area of the BC was quite limited (≈50 m^2^/g), which could explain the low level of SMZ adsorption onto the BC compared to other studies with larger surface areas, where a high adsorption of sulfonamides was observed through this mechanism [99,100]. Additionally, π-π interactions between the aromatic rings of the sulfonamide molecule and the functional groups on the BC may have contributed to the adsorption process, although to a lesser extent than in the case of FLX. Similarly, hydrogen bonding between the amine group of SMZ and the oxygenated groups on the BC, as mentioned above, could also have been involved.

Given the good results obtained for the cationic compound, the corresponding FLX adsorption isotherms with BC (Figure 3b) were calculated by determining the adsorption capacity according to Equation (S2) (Table S1). The experimental maximum adsorption capacity achieved for FLX was approximately 10.00 mg/g. Previous studies reached adsorption capacities of FLX above 90 mg/g with commercial adsorbents [101]. Román et al. [102] performed a hydrothermal synthesis of adsorbents based on a walnut shell, sunflower stalk and olive stone, obtaining maximum adsorption capacities of 22.43, 16.05 and 44.07 mg/g, respectively. Moreover, Silva et al. [97] employed pine bark, cork residues and spent coffee grounds for the synthesis of different BCs, obtaining maximum adsorption values ranging from 4.74 to 14.31 mg/g of FLX, in line with those obtained in this work.

The fitting was performed to the isotherm models include in Table S2: Langmuir (Equation (S5)), Freundlich (Equation (S6)) and Redlich–Peterson (Equation (S7)), whose setting parameters are shown in Table S3. The determination coefficients (R^2^) obtained were 0.951, 0.980 and 0.990, respectively, with the Redlich–Peterson model providing the best fit. The parameters associated with the model were k = 2.52 L/g, α = 0.17 (L/mg) ^β^ and β = 0.47. This model was a hybrid resulting from the Langmuir and Freundlich models, indicating that surface heterogeneity (β < 1) influenced the adsorption process [103,104], with sites that did not follow the ideal monolayer adsorption.

### 2.3. Enhanced Sulfonamide Adsorption

**Figure 4:**
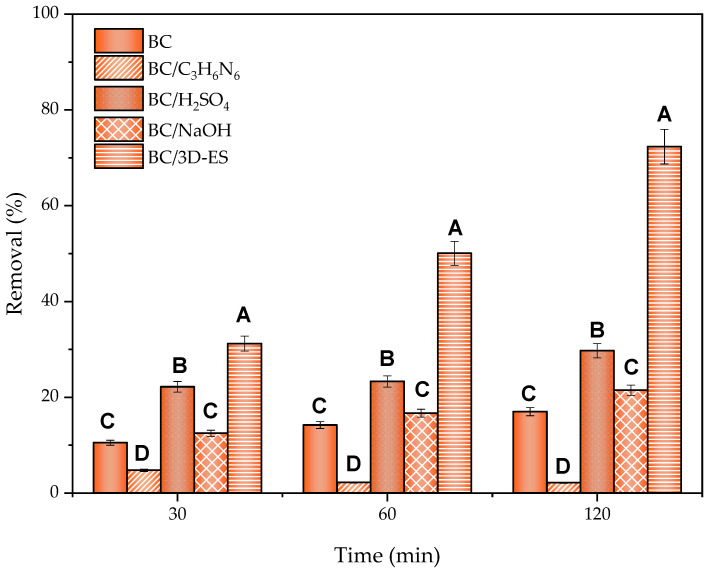
BC modifications for enhanced SMZ removal over 120 min of treatment with pollutant concentration of 10 mg/L and 10 g/L of adsorbent. BC was modified with 0.5 M C_3_H_6_N_6_; 2 M H_2_SO_4_; 2 M NaOH and electric field was applied at 1.2 V. Letters represent different groups of the studied factor (modifications) significantly different from each other in relation to the response variable (removal). The group’s influence is ordered decreasingly, with A being the highest impact group, followed by B, C and D.

Various modifications were made to the BC in order to improve the adsorption capacity of SMZ. For this purpose, the incorporation of new functional groups on the BC surface was sought by performing acid (BC/H_2_SO_4_) and basic (BC/NaOH) treatments, as well as nitrogen doping by hydrothermal treatment with melamine (BC/C_3_H_6_N_6_). The material surface was also modified by low-potential electric field-assisted adsorption or electrosorption (BC/3D-ES) treatment, as this was reported to be a method for improving pollutant removal [56]. Figure 4 shows the SMZ removal percentages achieved in each modification at different treatment times.

After 120 min of treatment, SMZ removal was reduced from the 17% obtained with BC without modification to 2% in the case of BC/C_3_H_6_N_6_. Although the addition of nitrogen groups in the BC would provide more electropositive behavior, it was already mentioned that electrostatic interactions did not represent a relevant factor for the adsorption mechanism. To verify this behavior, the zeta potential value was determined for the different modifications at working pH. The values are shown in Table 3:

As can be seen, the treatment with C_3_H_6_N_6_ was associated with the lowest electronegative value among the different modifications. Therefore, if the system behavior was conditioned by electrostatic interactions, this material should have exhibited the lowest repulsion toward the pollutant, which was in its negative form. Similarly, its proximity to the unmodified BC zeta potential value should also have implied a similar elimination, but this was not the observed situation. Therefore, the low percentage of pollutant removal could be explained by the binding of the amine group from C_3_H_6_N_6_ with the BC functional groups responsible for the π-π interactions and hydrogen bonds, reducing the SMZ access to active sites.

The BC/H_2_SO_4_ acid treatment resulted in a slight improvement in SMZ compared to pristine BC, reaching 30% removal. This result highlights again the absence of electrostatic interactions as the main mechanism involved, since this modification showed the most electronegative zeta potential value. Hence, this condition should be the least favorable for SMZ adsorption, while the results obtained indicated the opposite. Bogyta et al. [105] found in the FTIR an increase in band intensity associated with the formation of new carbonyl groups after the modification of wood biochar with H_2_SO_4_ at concentrations above 1 M, as well as the presence of new OH groups on the adsorbent surface. These changes could favor the pollutant adsorption on the material surface, enhancing the mechanisms mentioned above. However, a significant reduction in the presence of aromatic groups at high acid concentrations was detected in the same study, which could counteract a favorable adsorption effect, impeding better results.

The material adsorption capacity was also slightly increased after BC/NaOH treatment, reaching 21% SMZ removal, which implied a 1.3-times increase in comparison to removal achieved with the untreated BC. Similar results were reported by Jin et al. [106], who increased the adsorption capacity of As(V) by 3% after the alkaline activation of BC obtained from municipal solid waste, explained by the influence of new hydroxyl (-OH) and carboxylic (-COOH) groups on the material surface after activation. However, no significant improvement was achieved with the modification, since the same category was assigned to the BC through statistical analysis (group C).

According to statistical analysis, the most significant treatment for the improvement of pollutant removal capacity was surface modification by means of a 3D electrochemical electrosorption test, reaching 72% removal in 120 min. In this technique, BC particles can be polarized with a certain potential to form charged microelectrodes, increasing the surface area of the electrode and improving interactions between the BC and ionized species in the electrolyte solution [107,108]. In a similar study, Correia-Sá et al. [109] employed a BC from vineyard pruning residue to perform a 3D electrosorption treatment. This process allowed them to achieve total carbamazepine removal due to the incorporation of polarized microparticles.

Once the BC/3D-ES treatment was established as the most favorable for SMZ removal, the process was evaluated by performing tests with different working electrodes and adsorbent doses.

### 2.4. The 3D Electrosorption Method

#### 2.4.1. Electrode and Dose Effect

Carbonaceous materials offer multiple interesting properties for their application in electrochemical techniques due to their high surface area and electrical conductivity or stability, in addition to being low-cost and easily obtainable materials [108,110]. Hence, the use of different carbonaceous materials as working electrodes to form the 3D electrosorption configuration, together with the BC, was evaluated in this work.

**Figure 5:**
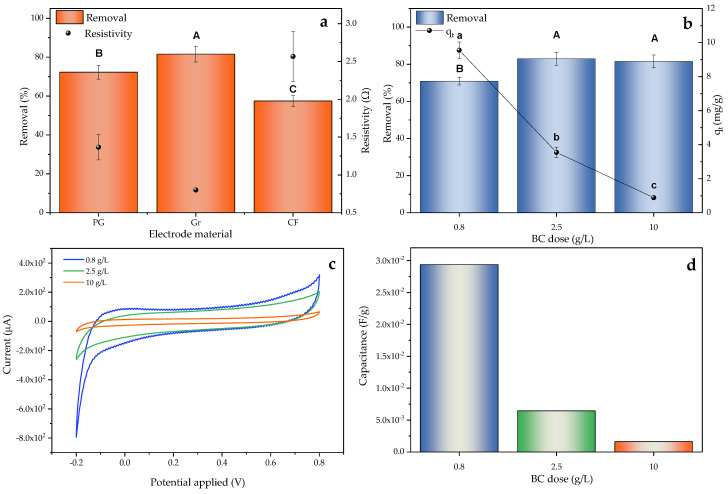
SMZ removal over 120 min according to (**a**) electrode material and (**b**) BC dose. (**c**) CV result after 5 cycles for doses of 0.8, 2.5 and 10 g/L BC. (**d**) Specific capacitance according to dose employed. Letters represent different groups of the studied factor (electrode effect and dose effect) significantly different from each other in relation to the response variable (removal and/or uptake). The group’s influence is ordered decreasingly. In case of removal, A is the highest impact group, followed by B, and C (if applied). In case of uptake, a is the highest impact group, followed by b and c.

The electrodes consisted of graphite paper (PG), carbon felt (CF) and graphite sheet (Gr). The SMZ removal percentages achieved at 120 min are detailed in Figure 5a. In this way, the removal efficiency followed the trend of Gr(+)Gr(−) > PG(+)PG(−) > CF(+)CF(−), in accordance with the allocation of groups A > B > C. This variation could be directly related to the resistivity (R) of the electrodes, since R(CF) > R(PG) > R(Gr), with CF exhibiting a resistivity 3.2 times higher than that of Gr.

The effect of the adsorbent dose is shown in Figure 5b. No change was observed after a 4-times reduction in the maximum adsorbent dose, with the removal rate remaining at around 80%. Thus, the amount of adsorbent was further reduced up to 0.8 g/L, where the removal decreased to 70%, but the adsorption capacity value was three times higher. This trend was well reflected in the statistical results. Although in terms of pollutant removal, the highest doses (10 and 2.5 g/L) were assigned to group A, in terms of uptake, it was the lowest level (0.8 g/L) that was allocated to this group.

This behavior, already observed in previous studies [111,112,113], indicated that the pollutant removal rate increased with the amount of adsorbent, due to the higher availability of active sites. However, after reaching an optimal dose, the removal no longer improved significantly, due to the overlapping and aggregation of active sites, impeding their effectiveness and stagnating or even reducing the adsorption rate. Likewise, with an optimal dosage value, the adsorption capacity increased significantly and reduced operating costs, so that in this work, this dosage was set at 0.8 g/L of BC.

The system behavior according to the used BC dose was also evaluated based on its electrochemical properties by means of cyclic voltammetry (CV) curves, shown in Figure 5c. The quasi-rectangular shape of the system and the absence of Faraday peaks in all cases indicated a performance close to the ideal electric double layer (EDL), typical of this type of system [114,115]. The obtained integration areas were directly related to the specific capacitance values, which are shown in Figure 5d and were calculated according to Equation (S3) (Table S1), where the best electrochemical yield was associated with the lowest dose, having a specific capacitance between 5 and 18 times higher than doses of 2.5 and 10 g/L of BC, respectively. This improvement could have been related to the higher effectiveness of electron transfer through the micro/mesopores at lower doses, since the agglomerations of material were avoided and the resistance in the medium was decreased, resulting in a higher specific surface area [116].

#### 2.4.2. Kinetic Study

Once the effects of different conditions of the BC/3D-ES were determined, a kinetic study of the process was conducted by adjusting the experimental data obtained to pseudo-first (PFO) and pseudo-second order (PSO) kinetics, according to Equations (S8) and (S9) of Table S4. Since, by definition, the adsorption capacity implies the presence of an adsorbent, in the Control 1.2 V test, this parameter was set to zero, so the fitting parameters listed in Table S5 only refer to the adsorption and BC/3D-ES processes.

**Figure 6:**
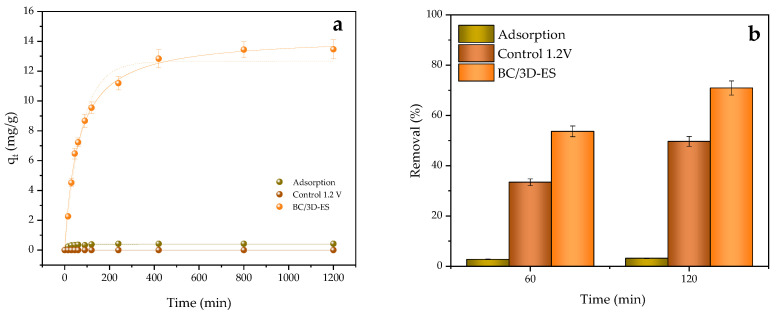
Profile of SMZ uptake over time and related removal with 3D electrosorption using 0.8 g/L of adsorbent to remove 10 mg/L of SMZ at 1.2 V. (**a**) Kinetic adjustment PFO (dotted line) and PSO (continuous line) and (**b**) synergistic effect by comparison of adsorption, control at 1.2 V and 3D electrosorption.

As shown in Figure 6a, the equilibrium conditions for BC/3D-ES were reached at around 6.5 h, with an uptake value of 12.84 mg/g, which increased slightly to 13.46 mg/g at 20 h. On the other hand, the experimental uptake at equilibrium for pure adsorption processes remained at 0.425 mg/g. In both cases, the R^2^ coefficients closer to the unit were obtained with PSO adjustment (0.983 and 0.995 for adsorption and BC/3D-ES, respectively), and there was a good agreement between the uptake values acquired experimentally and those reported by the model. This implied that the adsorption mechanism was controlled by the bond formation in the available sites of BC, rather than by the availability of sorbate in the solution.

Even though the adsorption capacity achieved by BC/3D-ES could still be improved compared to other studies, the relative enhancement provided by the modification was significantly higher, increasing 35-fold compared to the conventional adsorption process. For example, Ding et al. [117] reported an uptake of 154.16 mg/g for tetracycline adsorption on BC obtained from KOH-modified sludge, with a value 4.5 times greater than the unmodified BC. Ma et al. [118] also enhanced the adsorption capacity of tetracycline by 2.0-fold after modifying BC with thiourea, reaching 1364 mg/g. Meanwhile, Shao et al. [119] improved the equilibrium adsorption capacity of ciprofloxacin by 1.85-fold, reaching 5.14 mg/g, for a rice straw-derived BC modified with NaOH and KMnO_4_.

#### 2.4.3. Synergistic Electrosorption Effect

As the electric field contribution could not be determined in the kinetic study, Figure 6b shows the SMZ removal percentages achieved at 60 and 120 min of treatment for the adsorption, BC/3D-ES and electrochemical control processes.

As can be seen, there was a synergetic effect in the case of BC/3D-ES, since the percentage of elimination achieved exceeded the sum of those obtained in the adsorption process and the electrochemical control independently, reinforcing the hypothesis of microelectrode formation in the medium. In this system, an electrostatic induction occurred on the BC particles through the action of the electric field, where the oxidation–reduction reactions took place at both ends of the particles, significantly increasing the available area for chemical reactions. Furthermore, BC polarization also exerted an effect on the pore volume and functional groups on the surface, contributing to the increase in adsorption capacity [120,121]. A similar behavior was observed in the study developed by Puga et al. [121], where the removal of FLX was significantly increased due to the polarization of carbonaceous aerogels.

Electrochemical impedance spectroscopy (EIS) analysis was performed using a H_2_SO_4_ solution, both in the presence and absence of BC, to assess the material effect on the system’s electrochemical properties. The inclusion of BC between the electrodes led to a decrease in the arc radius in the Nyquist plot (Figure S1). This region is associated with the charge transfer resistance, so its reduction suggested an increase in the electrode transfer at the electrode/solution interface [122]. This behavior highlighted the contribution of the BC in the system even more, with it acting as a conductive material that improved the efficiency of the electrochemical process.

### 2.5. Regeneration and Reusability

Reuse capacity is a fundamental aspect when determining the adsorbent material viability, since the effective regeneration of BC allows for reduced operating costs and minimized environmental impacts. Thus, the material reuse capacity was evaluated in consecutive cycles, subjecting BC to different regeneration techniques: desorption with ACN and electro-regeneration.

**Figure 7:**
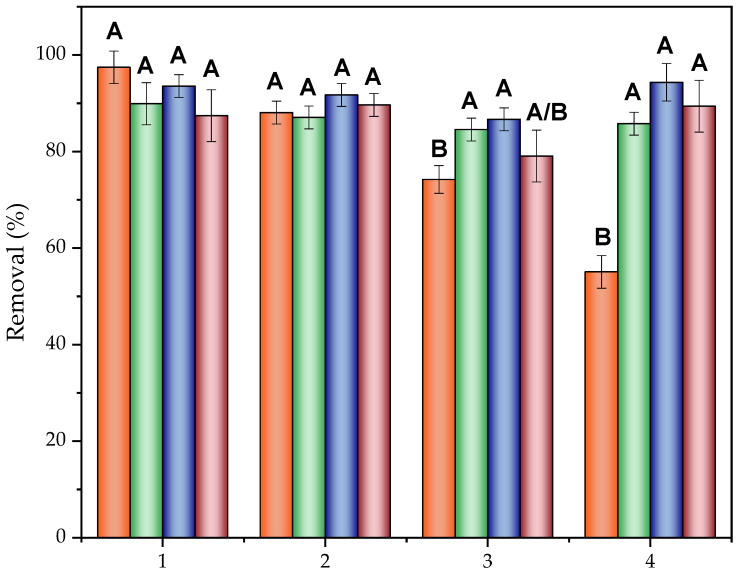
Consecutive cycles of use after BC regeneration through desorption with ACN (orange) and electro-regeneration at 25 mA (green), 50 mA (blue) and 100 mA (purple). Letters represent different groups of the studied factor (regeneration) significantly different from each other in relation to the response variable (removal). A and B are the highest and lowest impact groups, respectively. Same letter in different regeneration methods implies no significant difference in response. Group A/B in the 3th use at 100 mA implies the treatment is equidistant from both groups due to its standard deviation.

ACN was used in other studies as an organic compound for the desorption and/or extraction of sulfonamides [123,124,125] as well as other solvents to regenerate the BC through adsorption–desorption processes [126,127]. However, the use of solvents does not avoid the presence of pollutants and requires the adsorbate to have higher solubility to be successfully extracted from the material. In this section, as shown in Figure 7, the efficiency of pollutant removal after several cycles is compared through a desorption process and electro-regeneration treatments at different current intensities.

Thus, SMZ removal efficiency was reduced by 42% after four cycles of electrosorption in the desorption regeneration system, while this efficiency remained stable with the application of an electric field over the BC dispersed on an electrolyte solution, regardless of the applied intensity. This behavior could be explained by the degradation of the pollutant retained in the BC through the effect of reactive oxygen species (ROS) generated with the application of high potentials (>1.2 V). This process may have occurred through two possible pathways: direct degradation on the biochar surface or desorption followed by the electro-oxidation of SMZ [128], both occurring due to the action of the hydroxyl radicals produced by the oxidation of water at high current densities [129,130].

Regarding the current intensity applied, assignments to the same group, group A, indicated that there were no significant differences in its increase. Then, 25 mA was established as the most appropriate value due to the low energy requirement in comparison to the other intensities applied. The energy consumption values were calculated based on Equation (S4) (Table S1) and are shown in Table S6.

### 2.6. Ionic Strength

Salt ions are commonly found in real wastewater environments and their presence can affect the efficiency of pollutant electrosorption processes. Thus, competitive electrosorption tests were performed for the SMZ solutions in the presence of different ions (Cl^−^, NO_3_^−^, PO_4_^−^) at molar ratios of 1:1, 1:5 and 1:10. These ratios allowed us to see the influence of ionized compounds in the electrosorption process, maintaining concentrations that are usually present in wastewater, as in the case of hospital effluents [131].

**Figure 8:**
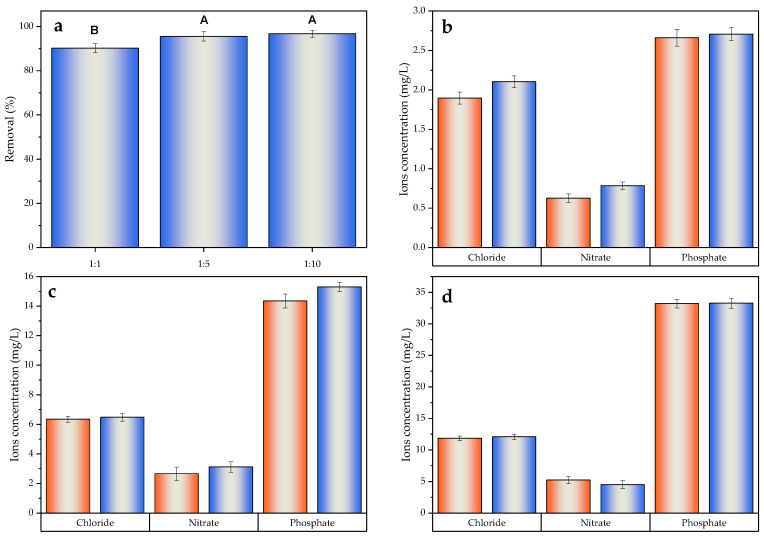
(**a**) SMZ removal after 24 h of BC/3D-ES treatment with different molar ratios of pollutant and ions. Letters represent different groups of the studied factor (molar ratio) significantly different from each other in relation to the response variable (removal). A and B are the highest and lowest impact groups, respectively. Same letter in different ratios implies no significant difference in response. Ion concentration for molar rates of (**b**) 1:1; (**c**) 1:5 and (**d**) 1:10, at initial time (orange) and after 24 h (blue) of treatment.

As can be seen in Figure 8a, the coexistence of anions in solution did not significantly affect the SMZ removal performance, reaching percentages above 90% regardless of the concentration used. Similar results were reported by Xu et al. [132] in the removal of Fl- in the presence of Cl^−^, AsO_2_^−^ and SO_4_^2−^ ions, which showed a removal close to 100% even at the highest ion concentration. This behavior was confirmed after ascertaining the concentration of ions present in solution (Figure 8b–d), which remained unchanged after treatment.

A slight increase in SMZ removal was observed as the ion coexistence increased, which was also reported after statistical analysis by integrating the ratios 1:5 and 1:10 into group A. This behavior could be justified by a salinization effect, where the solubility of compounds in aqueous solutions can vary in the presence of Na^+^ ions (resulting from the dissociation of NaCl, Na_2_SO_4_, Na_3_PO_4_ and NaNO_3_), increasing the activity coefficient of hydrophobic organic compounds [133,134], and consequently influencing hydrophobic interactions between the pollutant and BC.

## 3. Materials and Methods

### 3.1. Reagents and Materials

Model pollutants employed in the assays (SMZ and FLX) as well as Na_2_SO_4_, which was used as electrolyte with >99% purity, were provided by Sigma Aldrich, St. Louis, MO, USA.

The compounds used for BC modifications were C_3_H_6_N_6_ from Thermo Scientific and H_2_SO_4_ and NaOH obtained from Scharlau. The compounds used to evaluate ionic competitiveness were NaCl from Fisher Chemical, Na_3_PO_4_ from Prolab and NaNO_3_ from Merck, Rahway, NJ, USA. Mili Q water (18.2 MΩ∙cm) was used to perform all solutions.

Regarding the organic compounds employed for the desorption and analytical methods, HPLC-grade acetonitrile (ACN) and HPLC-grade methanol (MeOH) from Fischer, and 99% pure ammonium formate (Form) purchased from Thermo scientific, were used.

The BC was originally from *Pinus Pinaster* biomass residues, provided by the Department of Natural Resources and Environment Engineering (Universidade de Vigo, Apsin), and synthesized according to González-Prieto et al. [59].

### 3.2. Biochar Characterization

Adsorption–desorption isotherms, surface area and pore size were characterized according to the BET technique under a continuous N_2_ atmosphere at 77.35 K and using a Micrometrics ASAP 2020 analyzer (Micronetrics, Norcross, GA, USA). The morphological analysis of the material was completed with Scanning Electron Microscopy and Energy Dispersive X-ray spectroscopy (SEM/EDS) using a JEOL JSM6010LA microscope (JEOL Ltd., Tokyo, Japan).

The presence of functional groups in the material was analyzed by Fourier transform infrared spectroscopy (FTIR) using a Nicolet 6700 spectrophotometer (Thermo Fisher Scientific, Waltham, MA, USA) equipped with a DTGS KBr detector. The chemical structure of the material was also analyzed by Raman spectroscopy using a Horiba Jobin Yvon HR800UV spectrometer (Horiba, Kyoto, Japan). XRD was also applied to determine the material crystallinity and to identify the crystalline phases using a Siemens D5000 diffractometer system (Siemens AG, Karlsruge, Germany) with a graphite monochromator. Finally, the thermal stability of BC was analyzed by TGA in the presence of air, using a Setsys evolution 16/18 TG-DSC analyzer (Setaram, Caluire, France)

Regarding the composition of the material, EA of the material was performed to determine the presence of carbon (C), nitrogen (N), hydrogen (H), sulfur (S) and oxygen (O) by means of a Fisons EA1108 CHNS-O elemental microanalyzer. The metal content was also measured by inductively coupled plasma optical emission spectrometry (ICP-OES) using a Perkin Elmer Optima 4300 DV analyzer (PerkinElmer, Waltham, MA, USA).

All these analyses were performed through the CACTI service, University of Vigo, Spain.

The analysis of BC surface charge as a function of pH was performed by determining the zero charge point, following the methodology established by Gogoi et al. [135].

### 3.3. Experimental Set-Up

#### 3.3.1. Adsorption Assays

Adsorption tests of the BC with anionic (SMZ) and cationic (FLX) pollutants were performed without pH adjustment. The pollutant concentration was kept at 10 mg/L, while the BC dose was initially set at 10 g/L with a particle size of less than 0.25 mm. Adsorption tests were carried out by shaking in an IKA RCT basic plate at 450 rpm for 4 h until stabilization, with a volume of 50 mL and at room temperature.

#### 3.3.2. Biochar Property Modification

Once the BC behavior was known, modifications were made based on three methodologies:

- Incorporation of functional groups with C_3_H_3_N_6_.
  An autoclave hydrothermal treatment was performed by mixing 5 g of BC in a 60 mL 0.5 M solution of C_3_H_6_N_6_ [136]. The treatment was maintained at 220 °C for 12 h and the recovered material was washed 5 times. The modified BC, denoted as BC/C_3_H_6_N_6_, was dried overnight at 60 °C. Once the treatment was completed, adsorption tests of BC/C_3_H_6_N_6_ with SMZ were performed, keeping the conditions specified in Section 3.3.1, but reducing the contact time to 120 min.
- Acid–base modifications with sulfuric acid and sodium hydroxide.
  Acid and basic treatments were applied to the BC by using 2 M solutions of H_2_SO_4_ or NaOH. In both cases, the material was placed in contact with each solution by agitation for 5 h at 60 °C. The material was then recovered and washed with water until a neutral pH was obtained in the wash water (pH ≈ 7). Finally, the BC was left to dry overnight at 60 °C. As in the previous case, once the modified materials were obtained, which were named BC/H_2_SO_4_ and BC/NaOH, the corresponding adsorption tests were performed with SMZ.
- The 3D electrosorption (BC/3D-ES).
  Continuing with the methodology specified in Section 3.3.1, a 3D SMZ electrosorption test was carried out by incorporating an anode and a cathode of graphite paper obtained from Mersen to the system, with dimensions of 15 × 25 × 0.5 mm. The electrodes were immersed in the solution until a working area of 2.3 cm^2^ was reached, and a potential difference of 1.2 V was applied on them with a Hanmatek HM305 power supply. This potential was reported in previous works as the maximum applicable voltage without triggering secondary reactions such as water electrolysis, which could reduce the process efficiency [121]. To ensure conductivity throughout the solution, a 10 mM concentration of Na_2_SO_4_ was added as an electrolyte. This treatment will hereafter be referred to as BC/3D-ES, and also remained active for 120 min.
  Under these same conditions, the effect of using different carbonaceous materials in the electrodes (PG, CF and Gr provided by Mersen, Carbon-Lorraine and AliExpress, respectively) and different doses of BC (10 g/L, 2.5 g/L and 0.8 g/L) were evaluated. The choice of electrodes was made based on the material’s low cost, while the adsorbent dosage was adjusted to the minimum amount necessary to maintain high FLX adsorption following the behavior of adsorption isotherms.
  To determine the electrochemical properties of the material, a Metrohm Autolab potentiostat (Metrohm AG, Herisau, Switzerland) was used to perform the measurements and the Nova 2.1.7 software was used for data visualization. The cyclic voltammetry (CV) technique was used in a 0.5 M Na_2_SO_4_ solution, since it was the working electrolyte, with a potential window between −0.2 and 0.8 V, a scan rate of 0.1 V/s and a 0.002 V step. Likewise, electrochemical impedance spectroscopy (EIS) was applied in a 0.5 M H_2_SO_4_ solution in the frequency range from 10^6^ to 10^−2^ Hz, with a sinusoidal perturbation of 0.02 Vs. These conditions made it possible to obtain CV curves and spectroscopies free of noise and with clear signals.

The zeta potential of all modified BC samples was measured using a Litesizer DLS 500 particle size analyzer (Catalent Inc., Somerset, NJ, USA) by suspending 1.5 mg of BC particles smaller than 63 µm in 50 mL of distilled water.

### 3.4. Reusability and Regeneration

Consecutive experiments using the material were conducted. Each experiment was run for 24 h to ensure that the equilibrium point was reached, with a constant voltage of 1.2 V and a BC dosage of 0.8 mg/L. At the end of each cycle, two different regeneration strategies were evaluated.

#### 3.4.1. Desorption of Adsorbed Pollutant

Once the BC/3D-ES treatment was completed, the material was recovered and dried overnight at 60 °C. Then, a desorption process of SMZ was carried out by employing 0.8 g/L of BC in 50 mL of ACN and keeping the mixture in agitation for 120 min. Once finished, the material was recovered by centrifugation at 8000 rpm for 10 min and dried again for its new use.

#### 3.4.2. Electro-Regeneration

The BC/3D-ES, dried and with the SMZ adsorbed, was placed in 50 mL of a 10 mM Na_2_SO_4_ solution, stirring the mixture to avoid the deposition of carbonaceous material. Then, the regeneration of the material was performed by applying different current intensities (25, 50, 100 mA), continuing the treatment for 120 min and using the same electrodes as those employed in the BC/3D-ES assays. The material was then recovered and dried for a new cycle of use.

### 3.5. Coexistence of Ions

Ion competition assays with SMZ on BC/3D-ES were performed by evaluating different molar ratios between the pollutant and the concentration of a mixture of ions (1:1, 1:5 and 1:10). The influence of Cl^−^, NO_3_^−^ and PO_4_^−^ was studied by adding NaCl, NaNO_3_ and Na_3_PO_4_, respectively. The conditions employed were those determined as being optimal, that is, 0.8 g/L of BC in 10 mg/L of solution polluted with SMZ and Na_2_SO_4_ in excess (10 mM). The assay was kept in agitation for 24 h with an electric field of 1.2 V.

Ion quantification was performed by ion chromatography with the CACTI service using an Metrohm 940 Professional IC Vario ion chromatograph (Metrohm AG, Herisau, Switzerland) equipped with a conductivity detector.

### 3.6. Analytical and Statistic Methods

Pollutant concentrations were quantified by high-performance liquid chromatography (HPLC) using a Jasco LC-4000 HPLC (Jasco Inc., Tokyo, Japan) integrating a Diode array detector (DAD).

The separation column selected was the Kinetex 5 µm Biphenyl 100 A (150 × 46 mm) provided by Phenomenex. Regarding the elution conditions, a gradient mode was selected, starting from a ratio of 65% H_2_O, 30% MeOH and 5% Form, which was varied for 3 min up to a ratio of 35% H_2_O, 60% MeOH and 5% Form, then remaining constant for the following 15 min. The initial conditions were recovered 2 min later and the system was allowed to stabilize for 8 min until the method was finalized. The mobile phase flow rate was set at 1 mL/min, and the wavelengths used were 226 nm and 263 nm for FLX and SMZ, respectively.

The obtained data were subjected to statistical analysis using One-Way ANOVA and the mean values were compared using Tukey’s test at α = 0.05. The tests were performed in triplicate.

This statistical method allowed the factors which had a greater impact on the efficiency of the adsorption process to be established. For this purpose, different letters (A, B, C, a, b, c) were assigned to the groups with statistically significant differences. Thus, A and C represented the factors with the greatest and least effect on the response variable, respectively.

## 4. Conclusions

Based on the results obtained, it was concluded that the BC without modification showed a good capacity for removing FLX, but this was limited to 17% in the case of SMZ. Several modifications were made to improve these results, and it was found that acid and alkaline treatments provided some benefits, but they were much less pronounced than those observed with the BC/3D-ES treatment, which established electrosorption as being the most effective strategy. Thus, the working conditions were optimized, establishing the Gr electrode as the best alternative based on its low resistivity, and a dose of 0.8 g/L of BC as being optimal, allowing for a threefold increase in the material’s adsorption capacity without compromising its removal efficiency. The regeneration process also indicated a good potential for material reuse, highlighting the use of an electric field to maintain efficiency in consecutive cycles.

Although the influence of competitive ions on the electrosorption process was evaluated, the removal efficiencies established that there was no significant competitive effect, regardless of ion concentration. However, a slight increase in SMZ removal was detected as the concentration increased, which was attributed to a salting-out effect that could improve interactions between the pollutant and the BC.

Further development of the technique is still required to reduce treatment times and demonstrate its applicability on a larger scale under real conditions, supported by a techno-economic analysis. However, these findings present BC/3D-ES as a sustainable and promising technology for wastewater remediation.

## Figures and Tables

**Table 1:** BC characterization analysis.

**BET parameters**	
S_BET_ (m^2^/g)	49.64
S_EBET_ (m^2^/g)	32.76
S_MP_ (m^2^/g)	16.89
V_MP_ (cm^3^/g)	0.0068
**Elemental analysis parameters**	
Carbon (Wt %)	71.95
Oxygen (Wt %)	14.71
Hydrogen (Wt %)	2.71
Nitrogen (Wt %)	0.53
Ash (Wt %)	10.10
**Electrochemical parameters**	
Zero-point charge (pH_zpc_)	7.67

**Table 2:** Parameters of FLX and SMZ.

Compound	pK_a_ Acid	pK_a_ Basic	Log K_ow_	Ref.
FLX	-	10.06	1.57	[91]
SMZ	2.1	5.3	0.54	[92]

**Table 3:** Zeta potential associated with each modification.

Adsorbent Material	Zeta Potential (mV)
BC	−20.43
BC/C_3_H_6_N_6_	−21.10
BC/H_2_SO_4_	−38.10
BC/NaOH	−22.10

## Data Availability

Dataset available on request from the authors

## Supplementary Materials

The following supporting information can be downloaded at: https://www.mdpi.com/article/10.3390/molecules30071435/s1, Figure S1: In situ electrochemical impedance spectroscopy obtained in the presence and absence of BC; Table S1: Equations and units for adsorption capacity, removal, specific capacitance and energy consumption; Table S2: Equations, parameters and units for isotherm models; Table S3: Isotherm fitting parameters; Table S4: Equations, parameters and units for kinetic models; Table S5: Kinetic fitting parameters; and Table S6: Energy consumption for regeneration at different intensity levels.
