# Supplementary material for: Three-Dimensional Electrosorption for Pharmaceutical Wastewater Management and Sustainable Biochar Regeneration
Source: Molecules. 2025 Mar 24;30(7):1435. doi: 10.3390/molecules30071435 (PMC11990488; doi:10.3390/molecules30071435)
Supplement: Supplementary file 1 [file molecules-30-01435-s001.zip › molecules-3518159-supplementary.pdf]

Supplementary material

For

Three-dimensional electrosorption for pharmaceutical wastewater management and sustainable biochar regeneration

Nuria Bernárdez-Rodas[1], Emilio Rosales[1], Marta Pazos[1], Óscar González-Prieto[2], Luis Ortiz Torres[2], M. Ángeles Sanromán[1]*

CINTECX, Universidade de Vigo, Bioengineering and Sustainable Processes Group, Chemical Engineering Department, Campus Lagoas-Marcosende, 36310, Vigo, Spain; *nuria.bernardez@uvigo.gal (N.B-R); emiliorv@uvigo.gal (E.R); mcurras@uvigo.gal (M.P); sanroman@uvigo.gal (M.A.S)*

Department of Natural Resources and Environment Engineering, University of Vigo, 36005, Pontevedra, Spain; *osgonzalez@uvigo.gal (O.G-P); lortiz@uvigo.gal (L.O.T)*

* Correspondence: *sanroman@uvigo.gal (M.A.S)*

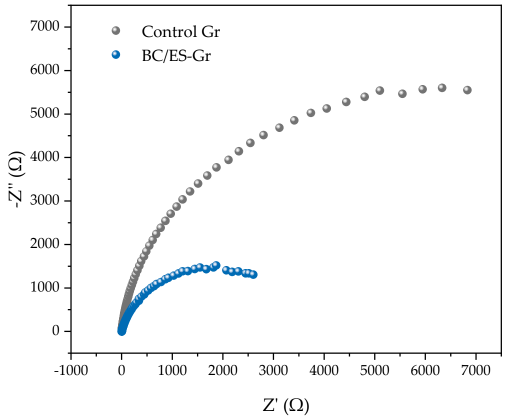

Figure S1. In situ electrochemical impedance spectroscopy obtained in presence and absence of BC.

Table S1. Equations and units for adsorption capacity, removal, specific capacitance and energy consumption.

Code	Parameter	Formula	Units
Eq. S1	Removal efficiency	$\text{Removal} = \frac{(C_0 - C_t)}{C_0} \cdot 100$	Removal (%)<br>C_0 (mg/L)<br>C_t (mg/L)
Eq. S2	Adsorption capacity	$q_t = \frac{(C_0 - C_t) \cdot V_{sol}}{m_{BC}}$	q_t (mg/g)<br>C_0 (mg/L)<br>C_t (mg/L)<br>m_{BC} (g)<br>V_{sol} (L)
Eq. S3	Specific capacitance	$C_s = \frac{\int_{V_{min}}^{V_{max}} IdV}{2 \cdot m_{BC} \cdot v \cdot (V_{max} - V_{min})}$	C_s (F/g)<br>I (A)<br>V_{max} (V)<br>V_{min} (V)<br>m_{BC} (g)<br>v (V/s)
Eq. S4	Energy consumption	$\text{EC} = \frac{V \cdot I \cdot t}{V_{sol}}$	EC (Wh/L)<br>V (V)<br>I (A)<br>t (h)<br>V_{sol} (L)

Table S2. Equations, parameters and units for isotherm models

**Code**	**Isotherm model**	**Isotherm formula**	**Units**
Eq. S5	Langmuir	$q_e = \frac{q_{max} \cdot k_L \cdot C_{eq}}{1 + k_L \cdot C_{eq}}$	q_e (mg/g)<br>q_{max} (mg/g)<br>k_L (L/mg)<br>C_{eq} (mg/L)
Eq. S6	Freundlich	$q_e = k_F \cdot C_{eq}^{1/n}$	q_e (mg/g)<br>k_F ($mg^{1-1/n} \cdot L^{\cdot 1/n} g^{-1}$)<br>$C_{eq}$ (mg/L)<br>n_F (dimensionless)
Eq. S7	Redlich-Peterson	$q_e = \frac{k_{RP} \cdot C_{eq}}{1 + \alpha_{RP} \cdot C_{eq}^{\beta_{RP}}}$	q_e (mg/g)<br>k_{RP} (L/g)<br>C_{eq} (mg/L)<br>α_{RP} $(L/mg)^{\beta_{RP}}$<br>β_{RP} (dimensionless between 0 and 1)

Table S3. Isotherm fitting parameters

Model	**Parameters**	**Value**	**R²**
Langmuir	q_{max} (mg/g)	9.064	0.951
	k_L (L/mg)	0.237	
Freundlich	k_F ($mg^{1-1/n} \cdot L^{\cdot 1/n} g^{-1}$)	2.241	0.980
	n_F	3.392	
Redlich-Peterson	k_{RP} (L/g)	2.521	0.990
	α_{RP} $(L/mg)^{\beta_{RP}}$	0.171	
	β_{RP}	0.473	

Table S4. Equations, parameters and units for kinetic models

**Code**	**Model**	**Formula**	**Units**
Eq. S8	Pseudo-first order (PFO)	$q_t = q_e \cdot (1 - e^{-k_1 \cdot t})$	q_t (mg/g)<br>q_e (mg/g)<br>k_1 (min^{-1})<br>t (min)
Eq. S9	Pseudo-second order (PSO)	$q_t = \frac{t}{\left(\frac{1}{k_2 \cdot q_e^2}\right) + \left(\frac{t}{q_e}\right)}$	q_t (mg/g)<br>q_e (mg/g)<br>k_2 (g/mg·min)<br>t(min)

Table S5. Kinetic fitting parameters

Model	Treatment	Parameters	Value	R^2
PFO	Adsorption	q_e (mg/g)	0.400	0.935
		k_1 (min^{-1})	0.048	
	BC/3D-ES	q_e (mg/g)	12.663	0.979
		k_1 (min^{-1})	0.013	
PSO	Adsorption	q_t (mg/g)	0.428	0.983
		k_2 (g/mg·min)	0.180	
	BC/3D-ES	q_t (mg/g)	14.355	0.995
		k_2 (g/mg·min)	0.001	

Table S6. Energy consumption for regeneration at different intensity levels

Intensity (mA)	EC (Wh/L)
25	9.49
50	18.98
100	37.96
